# Effect of sIL-13Rα2-Fc on the progression of rat tail intervertebral disc degeneration

**DOI:** 10.1186/s13018-019-1361-0

**Published:** 2019-11-27

**Authors:** Xin Wang, Junhao Sun, Jianshi Tan, Pengzhong Fang, Jinlei Chen, Wen Yuan, Huajiang Chen, Yang Liu

**Affiliations:** 1 Department of Orthopedics, First Clinical Medical College of Lanzhou University, The First Hospital of Lanzhou University, Lanzhou, 730000 Gansu China; 2 Changzheng Orthopedics Hospital, Second Military Medical University, Shanghai, 200003 China

**Keywords:** Intervertebral disc degeneration, sIL-13Rα2, Collagen, Fibrosis

## Abstract

**Background:**

The incidence of degenerative disc disease caused by intervertebral disc injury is increasing annually, seriously affecting the quality of life of patients and increasing the disease burden on society. The mechanisms of intervertebral disc degeneration include changes in extracellular matrix (ECM) deposition and tissue fibrosis. sIL-13Rα2-Fc potently inhibits interleukin (IL)-13, as well as blocks related cell signaling pathways and inhibits fibrosis in certain tissues. However, it is unknown whether sIL-13Rα2-Fc inhibits fibrosis in injured intervertebral discs and slows the process of degeneration. We hypothesized that sIL-13Rα2-Fc delays the progression of intervertebral disc degeneration by inhibiting intervertebral disc fibrosis and improving ECM deposition.

**Methods:**

A rat tail intervertebral disc degeneration model was established. Pathological changes in rat intervertebral disc tissue were observed by hematoxylin and eosin staining and Masson staining. Glycosaminoglycan (GAG), chondroitin sulfate (CS), keratan sulfate (KS), and hyaluronic acid (HA) contents were quantitatively analyzed by enzyme-linked immunosorbent assay. Type I and type II collagen expression levels were analyzed by reverse transcription-PCR and western blotting.

**Results:**

Hematoxylin and eosin staining and Masson staining revealed annulus fibrosus rupture, disordered arrangement, decreased nucleus pulposus tissue, and decreased collagen fiber in the rat intervertebral disc tissue. Following treatment with sIL-13Rα2-Fc, pathological changes in the rat intervertebral disc were reduced. Rat intervertebral disc tissue showed decreased GAG, CS-KS, and (HA) contents, increased type I collagen levels, and decreased type II collagen levels in degenerated intervertebral discs. sIL-13Rα2-Fc intervention increased the contents of GAG, CS, KS, and HA; inhibited the expression of type I collagen; and promoted the expression of type II collagen.

**Conclusion:**

These results demonstrate that intervertebral disc degeneration is associated with tissue fibrosis. sIL-13Rα2-Fc can regulate type I and type II collagen expression levels by increasing GAG, CS, KS, and HA contents, thereby slowing the progression of intervertebral disc degeneration.

## Introduction

Degeneration of intervertebral disc tissue may occur after injury, causing neurological disorders including low back pain. This condition affects approximately 5.4 million people worldwide, seriously decreasing their quality of life and increasing the disease burden on society [1]. The incidence of chronic back pain has increased by 54% since 1999 [2]. Many factors affect the development of low back pain, but the main cause is intervertebral disc degeneration [3].

The intervertebral disc is a cartilaginous joint between adjacent vertebral bodies and a complex structural tissue with multiple components. It is composed of annulus fibrous (AF) and nucleus pulposus (NP) tissue, and nutrients diffuse to the intervertebral disc through the cartilage endplates (CEP). The AF is primarily composed of fibroblast-like annulus fibrosus cells and type I collagen and synthesizes layered collagens, elastins, proteoglycans, and other non-collagen proteins. The NP is primarily composed of extracellular matrix (ECM) components such as type II collagen and proteoglycans which preserve moisture. Fibrous structures in the AF surround the gel-like NP to maintain the elasticity and mechanical strength of the intervertebral disc [4, 5].

Intervertebral disc degeneration is a complex, multifactorial process whose mechanisms involve tissue fibrosis, inflammatory responses, intervertebral disc malnutrition, changes in the ECM, natural aging, and cumulative injury [3, 6, 7]. Fibrosis is a basic pathological change that occurs in many chronic non-infectious diseases and is the main cause of disability and death in many chronic diseases; fibrosis affects nearly all organs and systems in the human body [8]. Excessive fibrosis can lead to excessive tissue remodeling that interrupts wound healing [9].

The development and progression of fibrosis is closely associated with immune effector cells and their released cytokines, among which interleukin (IL)-13 is one of the most important Th2 cytokines regulating fibrosis [10]. IL-13 exerts biological effects by binding to IL-13 receptors, including IL-13 receptor α1 (IL-13Rα1) and IL-13 receptor α2 (IL-13Rα2). IL-13Rα2 exists in three forms: a membrane-bound protein, intracellular protein, and soluble extracellular soluble protein, among which soluble IL-13Rα2 (sIL-13Rα2) plays a key role in the IL-13 response [11]. IL-13 binds to the IL-13Rα1 receptor complex on the surface of fibroblasts, resulting in phosphorylation of the signal transducer and activator of transcription 6 (STAT6). Phosphorylated STAT6 forms a homodimer in the cytoplasm and binds to gene transcriptional initiation sites in the nucleus, thereby promoting the transcription of collagen-related genes [12, 13]. Therefore, it has been suggested that administration of highly efficient antagonists of these cytokines may slow the degeneration process in intervertebral disc injury.

Previous studies have shown that sIL-13Rα2-Fc can effectively block the function of IL-13 in the process of fibrosis and reduce the deposition of abnormal ECM in injured tissues [14, 15]. However, whether sIL-13Rα2-Fc inhibits fibrosis in the injured intervertebral disc and slows degeneration is unknown. Therefore, to investigate whether sIL-13Rα2-Fc has therapeutic effects on intervertebral disc degeneration in rats, we used an annulus fibrosus puncture method to establish a rat model of intervertebral disc degeneration. We studied the protective effect of sIL-13Rα2-Fc on intervertebral disc degeneration and its mechanism by observing pathological changes and changes in the ECM in degenerated intervertebral disc tissue.

## Materials and methods

### Establishment of rat tail intervertebral disc degeneration model

Animal experiments performed in this study were approved by the Institutional Animal Care and Use Committee of the First Hospital of Lanzhou University, Gansu, China. The rats used in the study were 8–10-week-old Sprague-Dawley rats weighing 250–300 g and purchased from Lanzhou Veterinary Research Institute, Chinese Academy of Sciences (Lanzhou, Gansu, China). The rats were randomly allocated into five groups: blank group, model group, sIL-13Rα2-Fc low-dose group, sIL-13Rα2-Fc middle-dose group, and sIL-13Rα2-Fc high-dose group. The rat tail disc degeneration model was established as described by Chia-Hsian et al. [7].

### Hematoxylin and eosin staining

Intervertebral disc tissue was removed, fixed in 4% paraformaldehyde (P1110, Solarbio, Beijing, China) for 4–7 days, immersed in ethylene diamine tetraacetic acid (EDTA) for 2–3 weeks, and embedded in paraffin for sectioning. After the sections were deparaffinized with xylene and ethanol, they were stained with hematoxylin and eosin (H&E) (G1120, Solarbio). Pathological changes in the rat tail intervertebral disc tissue were observed under a light microscope.

The methods used to read or score the histological sections after H&E or Masson staining have been described by Mohd et al. [16]. Three observers evaluated the histological sections.

### Masson staining

The procedure was performed according to the instructions of the Masson staining kit (G1340, Solarbio). The slides were sealed with neutral resin and photographed under a microscope.

### Enzyme-linked immunosorbent assay analysis

Enzyme-linked immunosorbent assay (ELISA) kits were used to evaluate rat glycosaminoglycan (GAG) (mlbio, Shanghai, China, ml059570), chondroitin sulfate (CS) (mlbio, ml059167), keratan sulfate (KS) (mlbio, ml059586), and hyaluronic acid (HA) (gersionbio, QS42052) according to the manufacturer’s instructions. The optical density was measured at 490 nm using a microplate reader (iMark 19718, Bio-Rad Laboratories, Hercules, CA, USA).

### Reverse transcription-polymerase chain reaction

Total RNA was extracted from the tissues using an RNeasy Mini Kit (74104, Qiagen, Hilden, Germany). RNA content was measured at A260/280 using a microspectrophotometer. Reverse transcription and PCR amplification were sequentially performed according to the instructions of the QuantiNova Reverse Transcription Kit (205411, Qiagen) and QuantiNova SYBR Green PCR Kit (208054, Qiagen), respectively. Each group of experiments was performed in triplicate. The primer sequences were as follows:

Col I: forward: 5′-GGGCAAGACAGTCATCGAATA-3′; Reverse: 5′-GATTGGGATGGAGGGAGTTTA-3’; Col II: forward: 5′-TCAGGAATTTGGTGTGGACATA-3′; Reverse: 5′-CCGGACTGTGAGGTTAGGATAG-3′; GAPDH: forward: 5′-GTCTTCACTACCATGGAGAAGG-3′; Reverse: 5′-TCATGGATGACCTTGGCCAG-3′.

### Western blot analysis

Approximately 20 mg of frozen tissue sample was lysed with RIPA tissue lysis buffer (YZ-C1053, Applygen Technologies, Inc., Beijing, China). Total protein was extracted from the rat tail intervertebral disc tissue, and extracted protein was quantified using the bicinchoninic acid method (BAC). The primary antibodies used in the experiments were anti-collagen I antibody (Abcam, ab34710), anti-collagen II antibody (Abcam, ab34712), and anti-β-actin antibody (Abcam, Cambridge, UK, ab8227). The band of WB was subjected to optical density (OD) analysis using Image-Pro Plus software, and the relative expression of the target protein was calculated by the following formula:

$$ \mathrm{Relative}\ \mathrm{expression}\ \mathrm{of}\ \mathrm{target}\ \mathrm{protein}={\mathrm{OD}}_{\mathrm{target}\ \mathrm{protein}}/{\mathrm{OD}}_{\upbeta -\mathrm{actin}} $$

### Data collection

Rats were anesthetized with sodium pentobarbital (1%, 6 mL/kg), the tail of the rat was cleaned, and the limbs of the rats were fixed in the prone position. A 20G puncture needle was used to puncture the C7/8 and C8/9 intervertebral spaces and gently rotated by 360°. After 30 s, the needle was withdrawn to a depth of 3 mm. The sham control group was punctured similarly, but the intervertebral disc was not touched. After 1 week of successful puncture, different concentrations of sIL-13Rα2-Fc (0.5, 1.0, 2.0 mg/kg, *n* = 12) were injected at the same site in each group of rats. The sham operation and model groups were administered the same volume of normal saline. Rats were provided with drinking water ad libitum during the experiment. Rats were sacrificed at 2, 4, and 8 weeks after sIL-13Rα2-Fc intervention, and we collected intervertebral disc tissue for experiments.

### Statistical processing of data

The experimental data were analyzed using SPSS 19.0 software (SPSS, Inc., Chicago, IL, USA). Quantitative values were expressed as the mean ± SD. Comparisons between multiple groups were performed using one-way analysis of variance, and pairwise comparisons were performed using the least significance difference test. A *p* < 0.05 was considered to indicate a statistically significant difference.

## Results

### Effect of sIL-13Rα2-Fc on rat tail intervertebral disc tissue morphology

**Fig. 1:**
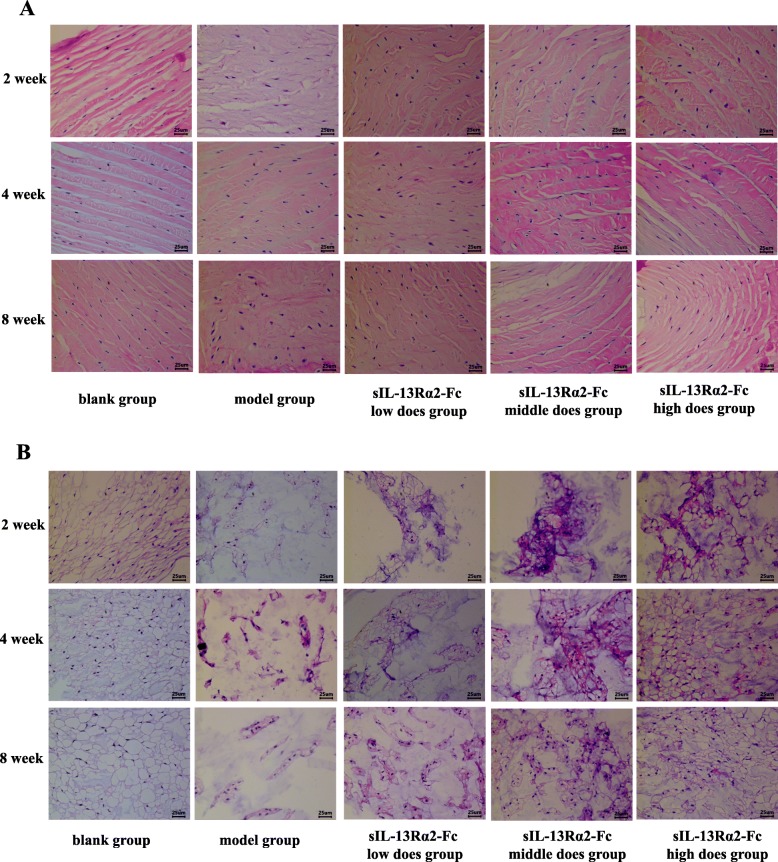
Hematoxylin and eosin (H&E) staining showing the histological effects of sIL-13Rα2-Fc intervention on intervertebral disc tissue in rats. **a** Histological assessment of annulus fibrosus in healthy and injured intervertebral discs (× 40). Compared with to the model group, sIL-13Rα2-Fc significantly slowed intervertebral disc degeneration in rats, which was characterized by uniform arrangement of the AF, reduction of the rupture site. **b** Histological assessment of nucleus pulposus in healthy and injured intervertebral discs (× 40). Compared with to the model group, sIL-13Rα2-Fc significantly slowed intervertebral disc degeneration in rats, which was characterized by increased number of NP cells

**Fig. 2:**
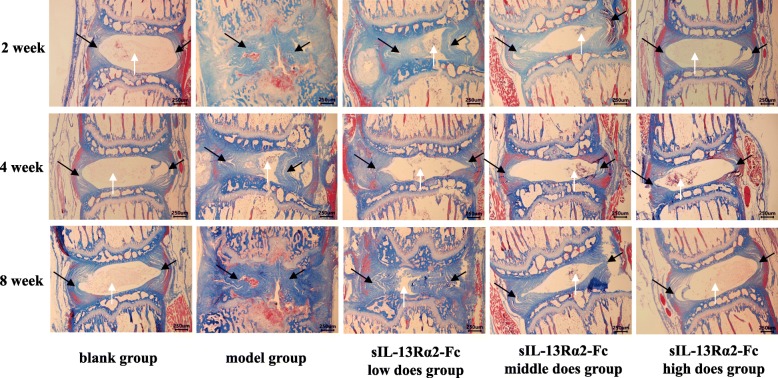
Masson staining showing the effects of sIL-13Rα2-Fc intervention on intervertebral disc degeneration in rats. There were varying degrees of damage in the intervertebral disc tissue in the model group, specifically presenting as AF rupture, disorganized arrangement, reduced number of NP cells, and a blurred boundary between the AF and the NP. The improvement of this damage was more obvious, with increasing of the dose of sIL-13Rα2-Fc intervention. AF: black arrow, NP: write arrow

We performed H&E staining analysis of rat intervertebral disc tissue at weeks 2, 4, and 8 of sIL-13Rα2-Fc intervention (Figs. 1 and 2). After successful model establishment, the number of NP cells in the intervertebral disc tissue of the model group and sIL-13Rα2-Fc intervention group was decreased, the arrangement of the AF was more disordered and exhibited ruptures, and the intervertebral disc tissue showed varying degrees of degeneration compared to the sham operation group. After 4 weeks of sIL-13Rα2-Fc intervention, the sIL-13Rα2-Fc (1.0, 2.0 mg/kg) groups exhibited improvements in intervertebral disc degeneration compared to in the model group, as observed by disordered arrangement and partial rupture of AF cells, a larger number of NP cells, and a widened junction between the AF and NP. After 8 weeks of intervention, the arrangement of AF in intervertebral disc tissues in the model group was even more disordered, the rupture site had expanded, the number of NP cells was decreased significantly, and degeneration was more obvious compared to in the sham operation group. Compared to the model group, sIL-13Rα2-Fc significantly slowed intervertebral disc degeneration in rats, which was characterized by uniform arrangement of the AF, reduction of the rupture site, and an increased number of NP cells. This demonstrates that sIL-13Rα2-Fc effectively slowed the progression of intervertebral disc degeneration. We graded the tissue sections according to the main subcategories of histological classification [16] (Table 1), recording scores of one for uninjured discs and higher scores in all categories for injured discs. We determined the total scores of injured intervertebral discs at 2 (Table 2), 4 (Table 3), and 8 (Table 4) weeks of sIL-13Rα2-Fc intervention.

**Table 1:** Histological grading criteria based on staining

Category	Score	Histological features
AF morphology	1	well-organized AF, half-ring-shaped structure, collagen lamellae
	2	Partly ruptured AF; loss of half-ring-shaped structure
	3	Completely ruptured AF; no intact half-ring-shaped Collagen lamellae
NP cellularity	1	Normal cellularity; no cell clusters
	2	Mixed cellularity; normal pattern with some cell clusters
	3	Mainly clustered cellularity; chondroid nests present
NP martix	1	Intense staining; blue staining dominates
	2	Reduced staining; mixture of blue and slight red staining
	3	Faint blue staining; increased red staining
Boundary between AF and NP	1	Clear boundary between AF and NP tissues
	2	Boundary less clear; loss of annular-nuclear demacation
	3	No distinguishable boundary between AF and NP tissues

**Table 2:** Mean total score of injured intervertebral discs after 2 weeks of sIL-13Rα2-Fc intervention

Groups	Score	Histological features
Blank group	4	The intervertebral disc is composed of AF arranged regularly around the periphery and the NP cells uniformly in the center. AF are arranged in half-ring-shaped structure. NP cells are distributed evenly, and the number of cells is larger.
Model group	11	There are fissures in the AF of the intervertebral disc, irregular arrangement. NP arrangement is obviously disordered, only part of the NP tissue is sparse ,and the number of NP cells is obviously reduced .
sIL-13Rα2-Fc low-dose group	9	The NP cells are irregular, the number of NP cells is less and the distribution of NP cells is more dispersed.
sIL-13Rα2-Fc middle-dose group	8	The boundary between the NP and the AF is clear,the number of NP cells begins to increase, and proteoglycan matrix begins to increase.
sIL-13Rα2-Fc high-dose group	7	The NP cells are further increased, cell distribution tends to be uniform, and arrangement of the AF tends to be neat.

**Table 3:** Mean total score of injured intervertebral discs after 4 weeks of sIL-13Rα2-Fc intervention

Groups	Score	Histological features
Blank group	4	The intervertebral disc is composed of AF arranged regularly around the periphery and the NP cells uniformly in the center.AF are arranged in half -ring -shaped structure .NP cells are distributed evenly ,and the number of cells is large.
Model group	12	The arrangement of intervertebral disc AF is more disordered and twisted. The NP cells are drastically reduced and unevenly arranged. The boundary between the AF and NP cells tends to be blurred and cracks appear.
sIL-13Rα2-Fc low-dose group	8	The AF ring of the intervertebral disc showed repair marks, and the arrangement of the AF tended to be neat, the number of NP cells was still small and unevenly distributed.
sIL-13Rα2-Fc middle-dose group	7	AF rings were arranged neatly, and degree of degeneration was gradually reduced. The NP cells increased, and the arrangment of parts AF were still disordered.
sIL-13Rα2-Fc high-dose group	6	Most of the AF rings are continuous and arrangement is very regular. The number of NP cells is large and evenly distributed. The boundaries of the AF and NP are clear, and the degree of degeneration is mostly reduced.

**Table 4:** Mean total score of injured intervertebral discs after 8 weeks of sIL-13Rα2-Fc intervention

Groups	Score	Histological features
Blank group	4	The intervertebral disc is composed of AF arranged regularly around the periphery and the NP cells uniformly in the center. AF are arranged in half-ring-shaped structure. NP cells are distributed evenly. and the number of cells is large.
Model group	12	There are fissures in the AF of the intervertebral disc, irregular arrangement. Nearly no boundary between the AF and the NP. and the NP cells almost disappear. replaced by AF tissue. showing fibrosis.
sIL-13Rα2-Fc low-dose group	7	The AF was repaired aud arranged neatly. NP cells gradually increased, and distribution gradually became uniform.
sIL-13Rα2-Fc middle-dose group	6	The number of NP cells is large. distribution is uniform, local disorder. AF arrangement is regular. and degree of degeneration continues to decrease.
sIL-13Rα2-Fc high-dose group	5	The NP cells are evenly distributed, increase in number. more uniformly arranged . And repair marks are visible in the AF ring, which are mostly coutinuous, the arrangement is regular, and degree of degeneration is severely reduced.

### Effects of sIL-13Rα2-Fc on GAG, CS/KS, and HA content of rat tail intervertebral disc tissue

**Fig. 3:**
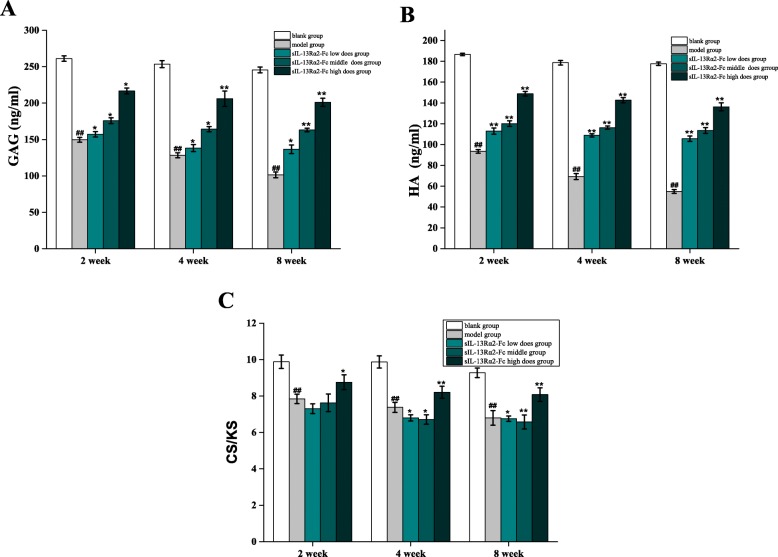
ELISA analysis of the effects of sIL-13Rα2-Fc intervention on glycosaminoglycan (GAG), hyaluronic acid (HA), and chondroitin sulfate/keratan sulfate (CS/KS) in rat intervertebral disc tissue. **a** sIL-13Rα2-Fc intervention increased the contents GAG levels in intervertebral disc tissue in each group. **b** sIL-13Rα2-Fc intervention increased the contents HA levels in intervertebral disc tissue in each group. **c** sIL-13Rα2-Fc intervention increased the contents CS/KS in intervertebral disc tissue in each group. ^#^*p* < 0.01, ^##^*p* < 0.05 compared to the blank group; *p < 0.01, **p < 0.05 compared to the model group

We performed ELISA to quantitatively measure the GAG, CS, KS, and HA contents of rat intervertebral disc tissue. In week 2 of sIL-13Rα2-Fc intervention, the GAG and HA contents in the model group were significantly lower than those in the sham operation group (*p* < 0.05), and the CS/KS ratio was also significantly lower (*p* < 0.05). The GAG content of the sIL-13Rα2-Fc (1.0, 2.0 mg/kg) groups was not significantly different from that of the model group (*p* > 0.05), GAG and HA contents were increased in the sIL-13Rα2-Fc (2.0 mg/kg) group (*p* < 0.01), and CS/KS ratio was increased significantly. The GAG and HA contents in the sIL-13Rα2-Fc (1.0, 2.0 mg/kg) intervention groups increased significantly over time compared to in the model group (*p* < 0.05), and the CS/KS ratio decreased slowly in a concentration-dependent manner (Tables 1, 2, and 3 and Fig. 3).

### Effect of sIL-13Rα2-Fc on type I and type II collagen mRNA expression in rat tail intervertebral disc tissue

**Fig. 4:**
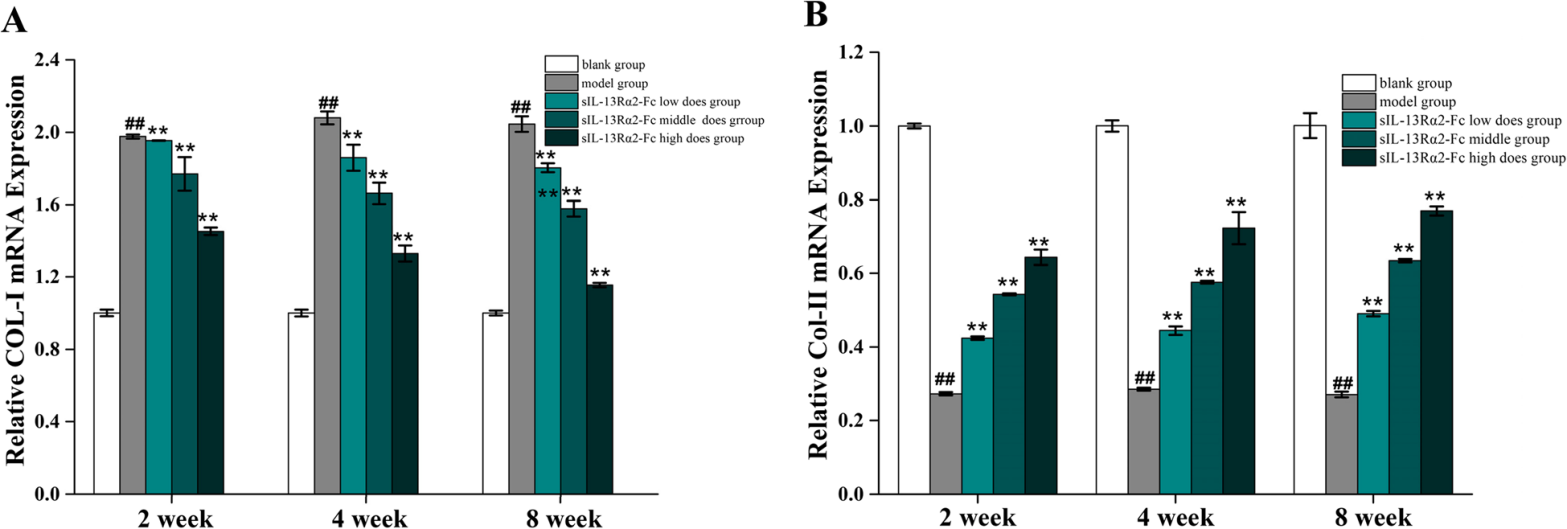
RT-PCR analysis of type I and type II collagen mRNA expression in rat tail intervertebral disc tissue. **a** sIL-13Rα2-Fc intervention inhibited the expression of type I collagen. **b** sIL-13Rα2-Fc intervention promoted the expression of type II collagen. ^#^*p* < 0.01, ^##^*p* < 0.05 compared to the blank group; *p < 0.01, **p < 0.05 compared to the model group

The process of intervertebral disc degeneration is accompanied by changes in gene expression. Reverse transcription-polymerase chain reaction (RT-PCR) was performed to evaluate the changes in type I and type II collagen mRNA expression levels in intervertebral disc tissue (Fig. 4). In degenerated intervertebral disc tissue, the expression of type I collagen was significantly increased (*p* < 0.05) and that of type II collagen was significantly decreased (*p* < 0.05). After 2 weeks of sIL-13Rα2-Fc intervention, there was no significant change in the expression levels of type I and type II collagen in the sIL-13Rα2-Fc (0.5, 1 mg/kg) groups (*p* > 0.05). As the duration of intervention increased, the expression level of type I collagen was significantly decreased (*p* < 0.05), whereas the expression level of type II collagen was significantly increased (*p* < 0.05) in a concentration-dependent manner.

### Effect of sIL-13Rα2-Fc on type I and type II collagen protein expression in rat tail intervertebral disc tissue

**Fig. 5:**
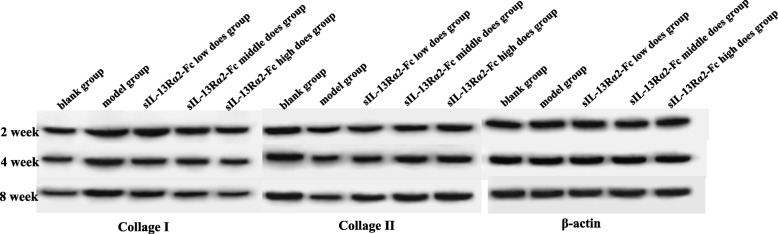
Western blot analysis of the changes in type I and type II collagen protein expression changes in intervertebral disc tissue. Compared with the model group, sIL-13Rα2-Fc intervention inhibited the expression of type I collagen, and promoted type II collagen expression

Western blot analysis was performed to determine the protein expression levels of type I and type II collagen in rat intervertebral disc tissue. Type I collagen protein levels were significantly higher (*p* < 0.05) and type II collagen protein levels were significantly lower (*p* < 0.05) in the model group than in the sham operation group. Compared to in the model group, there was no significant change in type I and type II collagen protein expression levels in the sIL-13Rα2-Fc (0.5, 1 mg/kg) intervention groups at 2 weeks after sIL-13Rα2-Fc intervention (*p* > 0.05). As the duration of intervention increased, the level of type I collagen protein significantly decreased (*p* < 0.05) and the level of type II collagen protein significantly increased (*p* < 0.05) in a concentration-dependent manner (Fig. 5).

## Discussion

Degenerative disc disease is a clinically common and frequently occurring disease. One of its characteristics is damage and lesions in peripheral tissues that do not heal easily. Pathological changes in the AF or cartilage endplates also indicate damage or inflammatory reactions in local tissues [3, 7]. Structural changes [17] in intervertebral disc tissue are hallmarks of impaired disc function, which are permanent structural changes. These structural changes are easily detected by physical and biological means [18, 19]. In the present study, we performed H&E and Masson staining to evaluate pathological changes in rat intervertebral disc tissue. The results revealed varying degrees of damage in the intervertebral disc tissue in the model group, specifically presenting as AF rupture, disorganized arrangement, reduced number of NP cells, and a blurred boundary between the AF and NP. After sIL-13Rα2-Fc intervention, this situation improved, indicating that sIL-13Rα2-Fc can alleviate the pathological changes associated with intervertebral disc degeneration.

Tissue damage can result in fibrosis or scar formation during repair. Tissue fibrosis primarily presents as an accumulation of large numbers of fibroblasts, ECM deposition, inflammatory reactions, and destruction of tissue structure [20]. Studies have shown that the development and progression of tissue fibrosis is affected by multiple factors, mainly an imbalance between the synthesis and degradation of collagen and the ECM [21, 22]. After the development of intervertebral disc disease, an imbalance between matrix synthesis and degradation is observed, resulting in changes in ECM composition and content [23, 24]. The ECM component of the intervertebral disc is primarily composed of collagen and proteoglycan [25]. After the development of intervertebral disc disease, changes in the proteoglycan content and composition are observed, which can directly lead to the decline or even loss of intervertebral disc biomechanical function, thereby causing a series of clinical symptoms [25, 26]. After the development of degeneration, proteoglycans are fragmented via the action of matrix-degrading enzymes, and small fragments are exuded from tissues, reducing the osmotic pressure of the intervertebral disc and weakening its hydrating effect. Additionally, the of proteoglycans, CS, KS, and HA are decreased [27]. Experimental studies showed that the protein content of degenerated intervertebral disc tissue is reduced, and the proteoglycan content can be used as an index of intervertebral disc degeneration [28]. In contrast, increased ECM decomposition can destroy the metabolism of intervertebral disc cells and synergistically promote intervertebral disc degeneration [29]. We conducted ELISA to quantitatively measure proteoglycans, CS, KS, and HA, and found that the content of these four ECM components was decreased to varying degrees in degenerated intervertebral disc tissue. This suggests that sIL-13Rα2-Fc has the therapeutic effect of slowing intervertebral disc degeneration.

Different degrees of fibrosis are observed in degenerated intervertebral disc tissue. Therefore, the specific mechanisms of fibrosis can be investigated by observing the expression characteristics of important factors of fibrosis. sIL-13Rα2-Fc is a potent inhibitor of IL-13, which can effectively block associated cell signaling pathways upon binding to IL-13, thus serving as a high-affinity antagonist of IL-13 for studies of interventions of IL-13 activity. For example, competitive inhibition of IL-13Rα1 binding to IL-13 leads to IL-13-mediated blockade of the IL-13/JAK/STAT6 signaling pathway, inhibition of abnormal collagen secretion and expression, and reduction of changes and deposition of the ECM, thereby reducing tissue damage [14, 30–32]. Belperio et al. [32] showed that sIL-13Rα2 downregulated the IL-13 content in the peripheral blood mononuclear cell culture medium of patients with schistosomiasis, as well as reduce tissue fibrosis. Lumsden et al. [33] showed that overexpression of sIL-13Rα2 in pulmonary fibrosis inhibited the expression of IL-13 and collagen, thus exerting an anti-fibrotic effect. Collagen is the principal ECM component of the intervertebral disc, and type I and type II collagen account for approximately 70% of the collagen in the intervertebral disc [34]. During intervertebral disc degeneration, the total amount of collagen in the tissue does not change significantly, but the type of collagen varies greatly, resulting in tissue abnormalities that occurs during degeneration and associated repair [35–37]. In intervertebral disc degeneration, abnormal collagen expression often occurs in fissures, blood vessels, and scar tissue, which alters the physiological characteristics and biomechanics of the intervertebral disc [38–40]. Antoniou et al. [28] observed increased synthesis of type I collagen and denaturation of type II collagen during intervertebral disc degeneration and fibrosis. In the present study, we injected sIL-13Rα2-Fc into intervertebral disc injury sites in rats and used RT-PCR to detect changes in type I and type II collagen expression. We found that type II collagen was decreased in the degenerated tissue and type I collagen expression was increased. sIL-13Rα2-Fc intervention reduced type I collagen mRNA expression and increased type II collagen expression in intervertebral disc tissue. This suggests that sIL-13Rα2-Fc can delay intervertebral disc degeneration to some extent; however, because of the limitations of the experimental conditions, imaging experiments were not conducted.

## Conclusion

Our results indicate that sIL-13Rα2-Fc can slow the progression of intervertebral disc degeneration to some extent in rats; improve pathological changes in intervertebral disc tissue; increase the contents of the ECM components GAG, CS, KS, and HA in tissues; inhibits the expression of type I collagen; and promote of the expression of type II collagen with sIL-13Rα2-Fc intervention. This study only confirmed that sIL-13Rα2-Fc can delay degeneration of rat intervertebral disc, but the specific mechanism is unclear. The next step is to use transcriptomics and proteomics techniques to screen for functional genes and marker proteins to obtain a comprehensive understanding of sIL-13Rα2-Fc on intervertebral disc degeneration and regulatory networks, providing a scientific basis for further clinical treatment.

## Data Availability

The datasets supporting the conclusions of this article are included within the article.

## Abbreviations

AF: Annulus fibrous
CS: Chondroitin sulfate
ECM: Extracellular matrix
GAG: Glycosaminoglycan
H&E: Hematoxylin-eosin
HA: Hyaluronic
IL-13: Interleukin-13
KS: Keratan sulfate
NP: Nucleus pulposus
OD: Optical density
RT-PCR: Reverse transcription-polymerase chain reaction
sIL-13Rα2-Fc: Soluble interleukin-13Ralpha2 receptor fusion protein
STAT6: Signal transducer and activator of transcription 6
